# Radiotherapy, EMP and dormancy in TME: An integrated perspective on tumor microenvironments and long-term relapse

**DOI:** 10.1016/j.isci.2026.116048

**Published:** 2026-06-09

**Authors:** Yebin Lee, Byeongseok Jang, Hae-Yun Jung

**Affiliations:** 1 Division of Radiation Biomedical Research, Korea Institute of Radiological and Medical Sciences, Seoul, South Korea

**Keywords:** radiation biology, molecular biology, cancer systems biology

## Abstract

Radiotherapy (RT) remains one of the most common modalities for curative treatment, with most cancer patients receiving RT during their treatment periods. Although there have been significant improvements in local targeting, local and distant recurrences after RT still remain a major clinical challenge. Dormant tumor cells, which are characterized by quiescent cells, are a potential driver of local recurrence and metastasis after RT. Epithelial-mesenchymal plasticity (EMP) is a central mechanism that allows disseminated tumor cells (DTCs) to enter a dormant state and reawaken to form recurrences years later. This review summarizes current knowledge of the molecular relationship between RT, EMP, and metastatic dormancy. We discuss the possibility whether RT modulates metastasis and dormancy. Ultimately, we propose promising therapeutic strategies to block RT-mediated metastasis and prevent long-term relapse in distant organs.

## Graphical abstract

**Figure:**
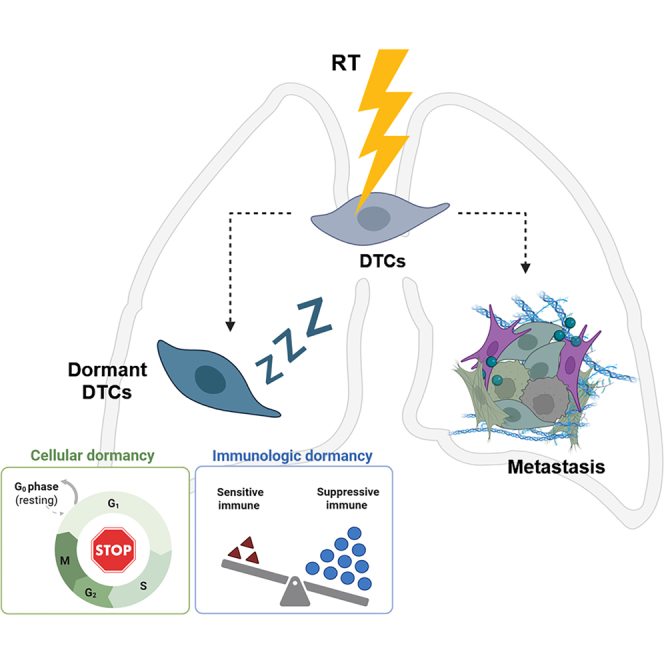

## Introduction

Radiotherapy (RT) has been the most effective cancer treatment that destroys cancer cells by causing DNA damage and promoting the generation of reactive oxygen species (ROS).^1^ Most cancer patients have RT as the standard treatment option, either as a primary treatment or as a combination treatment with surgery, chemotherapy, and immunotherapy.^2^^,^^3^^,^^4^ Despite its therapeutic success and usefulness, most patients suffer cancer recurrences and metastases after RT.^5^^,^^6^^,^^7^^,^^8^ In many cases, residual cancer cells after RT adapt to the surrounding environment for survival and acquire the capability of cell plasticity, motility, and treatment refractoriness.^9^^,^^10^

Recent studies suggest that RT affects directly irradiated cancer cells as well as the surrounding tumor microenvironment (TME), altering immune cell repositioning and extracellular matrix (ECM).^11^^,^^12^^,^^13^ TME reprogramming, such as ECM stiffness, is one of the triggers that provoke epithelial-mesenchymal plasticity (EMP).^14^^,^^15^^,^^16^ Additionally, EMP has been linked to cancer dormancy, a state of quiescent cell proliferation that enables cancer cells to promote therapeutic resistance and immune evasion.^17^^,^^18^ These dormant cancer cells can persist for several years, eventually reawakening to cause relapse or distant metastasis.^19^ The intersection of radiation-mediated EMP and metastatic dormancy provides a new paradigm for understanding distant recurrence and treatment resistance.

In this review, we aim to comprehensively explore the possible molecular links between RT, EMP, and metastatic dormancy and suggest the clinical and therapeutic approaches for RT to improve long-term control of RT-mediated metastasis.

## Radiotherapy

### RT in clinical relevance

RT is extensively utilized for a wide range of tumors, including lung, breast, cervical, and colorectal cancers.^20^ RT is used as a primary treatment for cancers, as adjuvant therapy after surgery to eliminate residual cancer cells, as neoadjuvant therapy before surgery to shrink tumors, and as palliative treatment to alleviate symptoms caused by cancer.^20^ Approximately 50% of cancer patients have received RT and 34% curative and 14% palliative response.^8^ RT provides 5-year local control benefit in 10.4% of all cancer patients and 5-year overall survival benefit in 2.4% of all cancer patients.^21^ These RT benefits depend on cancer types, ranging from the highest for cervical (33%) and head and neck cancers (32%) to no benefit for pancreas and colon cancer.^8^^,^^21^ The relatively low efficiency contributes to adioresistance, which results in local relapse, metastatic dissemination, and poor prognosis.

### Biological effects of radiation on tumors

#### Effect of radiation on cancer cells

**Figure 1:**
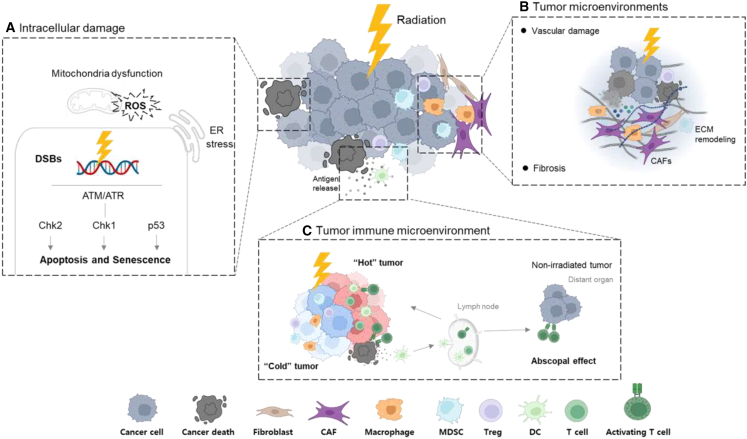
Radiation affects intracellularly and extracellularly to modulate tumor microenvironment (A) In the intracellular aspect, radiation directly induces DSBs through the ATM/ATR/DNA-PK cascade signaling pathways to promote cell death. Radiation also results in mitochondrial dysfunction to generate ROS and ER stress. Radiation-induced ROS activate the secretion of inflammatory cytokines to cause tumor microenvironments (TME) reprogramming. (B) In the TME aspect, radiation causes vascular damage and CAF-mediated ECM remodeling to cause fibrosis. (C) In the immune aspect, radiation-mediated numerous inflammatory cytokines play as dual role in TME reprogramming, for example, displaying immunosuppressive and immunosensitive features. Immunosuppressive cytokines, including TNF, IL-1β, IL-6, IL-10, and TGF-β, recruit locally suppressive immune cells such as TAMs, MDSCs, and Tregs. TGF-β also activates CAFs via ECM remodeling to cause fibrosis. Additionally, irradiated cancer cells release antigens, followed by the recognition of DCs and activation of T cells to attack cancer cells, known as the radiation-induced abscopal effect.

Radiation exerts direct cytotoxicity by inducing DNA damage and generation of ROS^11^^,^^22^ (Figure 1A). Radiation-induced DNA double-strand breaks (DSBs) result in apoptosis and senescence through activating canonical repair pathways such as ATM/Chk2, ATR/Chk1, DNA-PK, and p53 signaling.^23^^,^^24^^,^^25^ Radiation-induced ROS affect tumor necrosis factor α (TNF-α) signaling to activate inflammatory response to modulate the TME.^26^^,^^27^ Beyond DNA lesions, radiation affects various cellular compartments, such as mitochondrial dysfunction and endoplasmic reticulum stress.^28^^,^^29^

#### Effect of radiation on TME

Radiation profoundly remodels the TME through endothelial damage, fibrosis, and cytokine secretion, including transforming growth factor β (TGF-β), interleukin 1β (IL-6), and CXCL12, which reshape the extracellular milieu^9^^,^^30^^,^^31^ (Figure 1B). Endothelial cell dysfunction and apoptosis lead to inflammation and fibrosis in the TME after radiation.^32^ Radiation-induced inflammation excesses the deposition of ECM by activating the production of TGF-β, ultimately leading to fibrosis formation.^33^^,^^34^ Additionally, cancer-associated fibroblasts (CAFs) activated by radiation modulate ECM remodeling to promote fibrosis.^35^^,^^36^ In the tumor immune microenvironment (TIME), radiation-exposed TME exhibits a dual face in immuno-stimulatory and immuno-suppressive effects via altered production of inflammatory cytokines^37^^,^^38^^,^^39^ (Figure 1C). TNF-α, IL-1β, IL-6, 1L-10, and TGF-β recruit suppressive immune cells such as tumor-associated macrophages (TAMs), myeloid-derived suppressor cells (MDSCs), and regulator T cells (Tregs). Conversely, damage-associated molecular patterns (DAMPs) and their corresponding pattern recognition receptor (PRR) signaling in irradiated tumors cause immunogenic cell death (ICD) that results in activating dendritic cells (DCs) and cytotoxic T cells. Furthermore, activating T cells affects non-irradiated tumor cells at distant organs, which has been termed as the abscopal effect. These radiation-induced abscopal effects inhibit metastasis, either with radiation alone or in combination with immunotherapy.^40^^,^^41^^,^^42^

## EMP and metastatic dormancy

### EMP and metastasis

**Figure 2:**
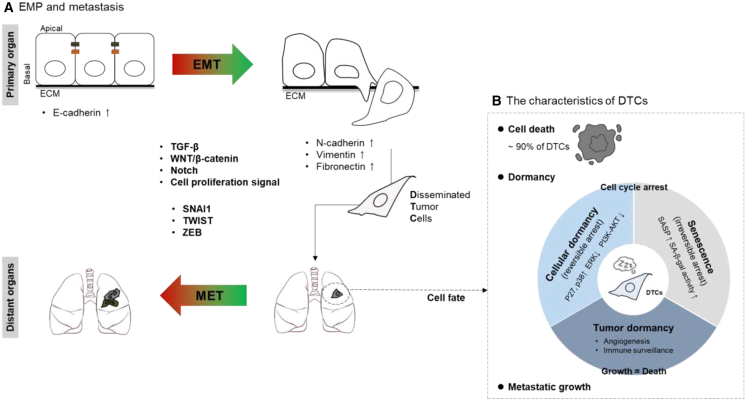
EMP is closely related to metastatic growth and dormancy (A) EMP and metastasis. EMT is a process by which epithelial cells acquire the capabilities of migration and invasion via the loss of epithelial cell polarity and cell-cell junctions. The EMT phenotype represents EMT markers such as the downregulation of E-cadherin and the upregulation of N-cadherin, vimentin, and fibronectin. MET is a reversible process of EMT in which mesenchymal cells lose their mobility and gain epithelial-like features. The EMP plasticity is regulated by multiple complexes with extracellular signaling such as TGF-β, WNT/β-catenin, Notch, and cell proliferation signals, and EMT TFs such as SNAI1, TWIST, and ZEB. (B) The characteristics of DTCs. DTCs determine cell death, cancer dormancy, and metastasis according to their surrounding environments. DTCs enter a dormant state to stop cell proliferation caused by the following: (1) senescence-irreversible cell cycle arrest with indicators such as SASP and SA-β gal activity, (2) cellular dormancy-reversible cell cycle arrest with indicators such as p27 and p38, and (3) tumor dormancy, which means equal cell growth and death, controlled by angiogenesis dysfunction and immune surveillance.

Epithelial-mesenchymal transition (EMT) is a cellular program in which epithelial cells lose cell-cell junctions and apical-basal cell polarity to gain mesenchymal characteristics such as cell mobility and invasiveness.^43^ This cellular phenotype is defined by upregulation or downregulation of typical EMT markers such as the downregulation of E-cadherin and the upregulation of N-cadherin, vimentin, and fibronectin.^44^ Mesenchymal-like cells can reverse their epithelial phenotype by undergoing a mesenchymal-epithelial transition (MET).^45^ EMT is regulated by multiple signaling pathways, including TGF-β, Wnt/β-catenin, Notch, PI3K/AKT, and MAPK signaling cascades.^46^ EMT transcription factors (EMT-TFs) such as SNAI1, TWIST, and ZEB are core regulators in these pathways^47^ (Figure 2A). EMP describes the cellular phenotype that allows cells to undergo reversible conversion between epithelial and mesenchymal states. This plasticity is crucial for metastasis formation, as cancer cells in the primary tumor disseminate to distant organs during the EMT process, while disseminated tumor cells (DTCs) in distant organs grow to form metastases during the MET process.^48^ Many cancer patients, including those with breast and colorectal cancers, have been observed to show metastasis a few years after cancer treatments.^49^^,^^50^

### EMP and metastatic dormancy

EMT-undergoing tumor cells disseminate to distant organs for survival and proliferation. Among DTCs, over 90% die and less than 10% adapt to survival in distinct organs. EMT-DTCs exhibit reduced proliferation, which facilitates their entry into dormancy, with less than 0.1% of DTCs entering the dormant state.^51^^,^^52^ Cancer dormancy is a process in which the cells enter a reversible state of cell cycle arrest, known as quiescence. DTC dormancy is maintained as either cellular dormancy or tumor dormancy. Cellular dormancy is a reversible growth arrest through cell cycle arrest and slow proliferation. These dormant cells exhibit reduced proliferation and metabolic activity and increased levels of CDK inhibitors and dormancy-associated factors such as p27 and p38. In contrast to cellular dormancy, senescence is irreversible growth arrest. Tumor dormancy is an equal state between cell proliferation and cell death, regulated by blood supply “angiogenic dormancy” and activating cytotoxic immune system “immunogenic dormancy”^53^^,^^54^^,^^55^ (Figure 2B). Dormant DTCs have the potential capability of metastatic relapse. Awakening of dormant cells often coincides with MET conversion. MET program allows cell to undergo proliferation to form metastasis in distant organs.^56^

Many cancer types exhibit partial or hybrid EMP states, expressing epithelial and mesenchymal markers simultaneously.^57^^,^^58^^,^^59^^,^^60^ EMP enables tumor cells to dynamically shift between states depending on the surrounding microenvironment and stimuli.^61^ EMP is intricately linked to cancer dormancy.^62^^,^^63^^,^^64^ Dormant states allow cells to adapt to stress and survive in a hostile microenvironment. Dormancy enables reawakening to promote cell proliferation to form recurrence or metastasis.^65^^,^^66^^,^^67^^,^^68^ Clinically, dormant states evade immune surveillance, resist cancer therapeutic treatments, and delay relapse.^69^

EMP also imparts cancer cells with stem cell-like characteristics, including self-renewal and differentiation capabilities.^70^^,^^71^^,^^72^ These cancer stem-like cells (CSCs) are frequently quiescent, drug resistant, and possess the capability to regenerate heterogeneous tumor populations.^73^ However, dormant cells and CSCs are not equal, as dormant DTCs may or may not acquire CSC feature.^74^

### Molecular link between EMP and metastatic dormancy

#### EMP-dependent metastatic dormancy

**Figure 3:**
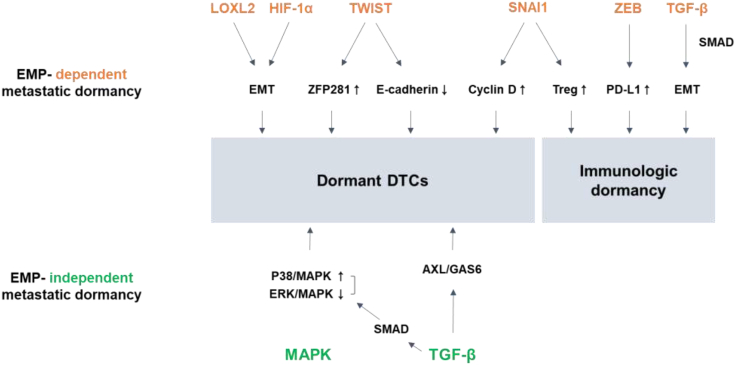
Molecular mechanisms of EMP and metastatic dormancy In EMP-dependent metastatic dormancy, EMT TFs such as TWIST1 and SNAI1 modulate dormant DTCs in distant organs. SNAI1 and ZEB control immune suppressive regulators such as Tregs and PD-L1, respectively, to contribute to immunologic dormancy. HIF-1α and LOXL2, regulators of the TME, cause dormant DTCs through the EMT process. In EMP-independent metastatic dormancy, MAPK signaling induces dormant DTCs via high p38/MAPK and low ERK/MAPK. Interestingly, TGF-β/SMAD signaling induces dormant DTCs and immune surveillance regardless of EMP dependence/independence. TGF-β also causes dormant DTCs via the AXL/GAS6 axis.

EMT TFs and the tumor microenvironmental factors, such as growth factors and hypoxia, lead tumor cells to enter a dormant state. SNAI1 induces cell cycle arrest through repressing cyclin D to result in low cell proliferation.^75^^,^^76^ TWIST1-repressed E-cadherin or -induced ZFP281 triggers cancer dormancy to prevent lung metastasis.^64^^,^^77^ Additionally, EMT TFs have been linked with immunogenic dormancy through immune inhibition.^78^ SNAI1-induced EMT activated Tregs and impaired DCs to escape the cytotoxicity of immune cells.^79^ ZEB-1/microRNA-200 suppressed immune response through upregulation of PD-L1 expression.^80^ Additionally, TGF-β, mainly derived from the TME, emerged as a primary driver of EMT process through increasing the expressions of SNAI1, ZEB, and TWIST.^81^^,^^82^ TGF-β evaded immune surveillance via inducing EMT in lung metastatic dormancy.^83^ TGF-β2 induced dormant DTCs through activating p38 in bone marrow or lungs.^84^^,^^85^ TGF-β/SMAD pathway was reported to regulate EMT and dormant tumor cells via different molecular mechanisms.^86^ Hypoxia-related factors such as hypoxia-inducible factor 1α (HIF-1α) and lysyl oxidase-like 2 (LOXL2) activates EMT process as a result of the dormant stage of tumor cells^87^^,^^88^^,^^89^ (Figure 3).

#### EMP-independent metastatic dormancy

Cell proliferation signaling pathways, including MAPK pathway, have also been associated with cancer dormancy. Dormant cells show low levels of ERK MAPK and high levels of p38 MAPK expression in various cancer types including breast cancer.^90^ Activating p38 MAPK allows DTCs to enter the dormant state through cell cycle regulators.^91^ TGF-β2 induces dormant DTCs via activating p38 singling in the bone marrow.^84^ TGF-β2 also causes prostate cancer dormancy in bone marrow through the AXL/GAS6 axis^92^ (Figure 3). Although bone morphogenetic proteins (BMPs), IFN-γ, and IL-6 are also related to cancer dormancy with various counterparts, the understanding of these signal-mediated dormant DTCs is still lacking.^53^^,^^90^

## Crosstalk between RT, EMP, and cancer dormancy

**Figure 4:**
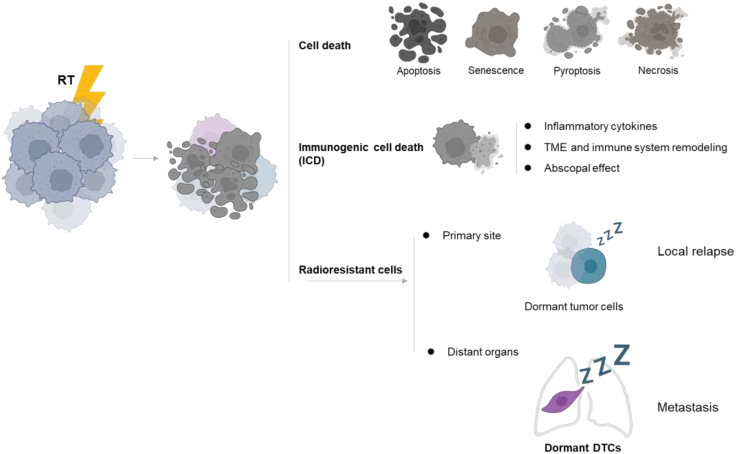
The anti-cancer effect of radiotherapy in clinical implication Irradiated tumors mostly confer cell death such as apoptosis, senescence, necrosis, and pyroptosis. Cancer cells undergoing death release numerous antigens and cytokines to modulate the TME and immune system. For example, radiation-induced ICD contributes to the frame transition from local treatment of radiotherapy to systemic treatment with immunotherapy. After radiotherapy, a few cells still survive to acquire dormant features to cause local or distant relapse later.

RT results in death of most cancer cells, followed by the release of a ton of inflammatory cytokines for TIME reprogramming and ECM remodeling. However, a few cells survive even after RT and reconstruct the TME. These cells might have the capacity for being dormant, leading to long-term relapse (Figure 4). In this section, we focus on whether RT induces metastasis through EMP and whether RT either awakens DTCs or keeps them dormant to control metastasis and prevent long-term relapse.

### RT and metastasis

Clinical observations suggest conflicting evidence that RT induces local pro-invasive and pro-metastatic capability by changing the local microenvironment, and, on the other hand, enhances the anti-metastatic effect and a systemic anti-cancer effect termed the abscopal effect.^4^^,^^8^^,^^93^^,^^94^ In line with this finding, preclinical studies have shown that the anti-cancer effect of RT is controversial. Many researchers have used various mouse models such as cancer cell types, inoculated sites, and different host immune systems to investigate whether radiation affects metastasis.^93^ Some studies have suggested that radiation significantly suppresses metastasis; conversely, other studies have revealed that radiation significantly increases metastasis through various signals and external stimuli^40^^,^^95^^,^^96^^,^^97^^,^^98^^,^^99^^,^^100^^,^^101^^,^^102^^,^^103^^,^^104^^,^^105^^,^^106^^,^^107^^,^^108^^,^^109^ (Table 1). Interestingly, radiation-induced EMT promotes metastasis in various cancer types. Molecular mechanisms underlying radiation-induced metastasis via EMT, in which TGF-β secreted from irradiated tumors promotes metastatic cancer progression through the EMT process.^97^ Radiation-induced CAFs enhance cancer metastasis through the production of TGF-β and inflammatory cytokines, mediating the EMT process.^109^^,^^110^ Additionally, radiation enhances SNAI1-mediated EMT process to trigger tumor cell invasion through the PI3K/AKT signal.^111^ However, the entire process of RT-mediated metastasis is not fully understood yet.

**Table 1:** The effect of radiation on metastatic capability

Organ	Cell lines	Inoculation site	Mouse strains	Radiation dose	Irradiated sites	Anticancer effect	Central regulators	Ref.
Breast	67NR	subcutaneous	balb/C	2 or 6 Gy	tumor	inducing abscopal effect	Flt3-ligand	Demaria et al.^40^
	MDA-MB-468 radioresistance cells	intracardiac	NSG	50 Gy for radioresistant: 2 Gy × 5, 4 Gy × 3, 6 Gy × 3, and 10 Gy × 1 for 6–8 weeks	cells	increasing metastasis	Integrin β3	Ghannam et al.^94^
	4T1 radioresistance cells	mammary gland	balb/C					
	4T1 or MDA-MB-231	mammary gland	nude	20 Gy	tumors	increasing metastasis	GM-CSF	Kumar et al.^95^
	MMTV/PyVmT/Luc cells	mammary gland	FVB	10 Gy	thoracic	increasing metastasis	TGF-β	Vilalta et al.^96^
	D2A1	mammary gland	balb/C	6 Gy × 4	pre-treat in mammary gland	increasing metastasis	IL-6, COX-2	Biswas et al.^97^
Bone	LM8	subcutaneous or intravenous	C3H/HeJ	1, 5 Gy	cells	decreasing metastasis	MMP-2, Ingergin αvβ3	Bouchard et al.^98^
Colon	HCT116	subcutaneous	nude	10 or 20 Gy	tumor	inducing abscopal effect	p53	Ogata et al.^99^
	SW480	subcutaneous	SCID	2 Gy	tumor	increasing metastasis	Macrophage	Strigari et al.^100^
Glioma	C6L	subcutaneous	balb/C	10 Gy per day for 5 days	tumors	increasing metastasis	EMT	Timaner et al.^101^
Liver	MHCC97L	liver	nude	2 Gy per 10 days	tumor in liver	increasing metastasis	TMPRSS4-EMT	Park et al.^102^
Lung	LLC1	subcutaneous	C57BL/6	20 Gy	flank tumors	increasing metastasis	Amphiregulin	Li et al.^103^
	LLC-LM and T241	subcutaneous	C57BL/6	10, 20, 30, 40, and 50 Gy	tumors	increasing metastasis	–	Piffko et al.^104^
	H1299	intravenous	nude	2 Gy	cells	increasing metastasis	HIF-α/CXCR4	Camphausen et al.^105^
Pancreas	MIAPaCa-2, BxPC3, Panc-1	intraperitoneal for liver metastasis	nude	2 and 5 Gy	cells	decreasing metastasis	Exosomal miR-3160-5p	Gu et al.^106^
	Panc-1	intravenous	BALB/c nude	4, 8, and 12 Gy	cells	increasing metastasis	HMGB1-EMT	Nakaoka et al.^107^
	Panc-1+CM from CAF with IR	intravenous	BALB/c nude	4 Gy	CAFs	increasing metastasis	CXCL12-CXCR4-EMT	Chen et al.^108^

GM-CSF, granulocyte-macrophage colony stimulating factor; IL-6, interleukin 6; COX-2, cyclooxygenase-2; MMP-2, matrix metalloproteinase-2; TMRPSS4, transmembrane serine protease 4; SCID, severe combined immunodeficiency; NSG, NOD/SCID/IL2Rγnull mice; FVB, friend virus B-type.

### RT and metastatic dormancy

Studies about the relationship between radiation and cancer dormancy are barely a few. RT induces the dormancy of cancer cells, and inflammation awakens dormant cells in intracerebral melanoma.^112^ Residual tumor cells after RT enter a “radiation-tolerant persister” (RTP) state as cancer dormancy and these RTP cells have phenotypic plasticity and tumorigenicity independent of stemness.^113^ Conversely, one review suggests the possibility that dormant cells with stemness awaken after RT to cause metastatic relapse in oral cancer.^114^ Current studies are too limited to determine whether RT induces tumor cells to enter the dormant state or awakens the dormant cells. Additionally, while RT causes metastatic cancer progression, it remains unclear whether the dormant DTCs exist during the metastatic process. Further studies should clarify the following as molecular mechanisms to prevent metastasis after RT: (1) whether RT induces dormant DTCs for long-term relapse, and (2) whether RT activates dormant DTCs to cause metastasis.

While CSCs are not equal to dormant cells in many features, radiation has been shown to induce CSC properties such as differentiation, self-renewal, and tumorigenicity in many cancer types.^74^ After radiation, surviving cells have both EMT and CSC properties, with high expression of SNAI1, CD24, CD44 in non-small cell lung cancer (NSCLC).^115^ Radiation also activates PI3K/AKT and p38 MAPK signals to acquire the ability of stemness and EMT progression in breast cancer.^116^ The above studies have revealed that CSCs gain radioresistance after RT in primary tumor, and there is no evidence for the role of CSCs in radiation-mediated metastasis.

## Therapeutic perspectives

**Figure 5:**
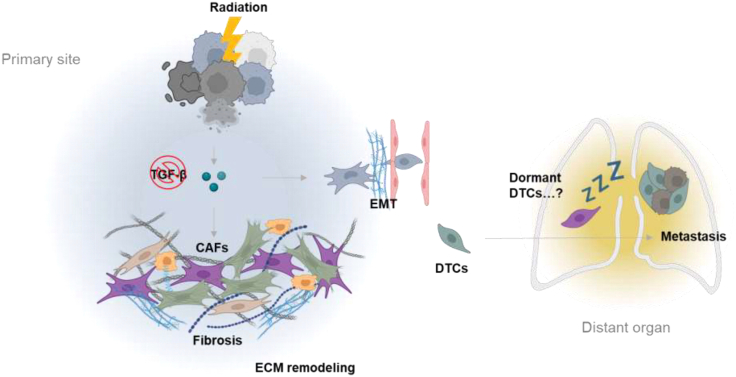
The possible biological function of TGF-β after RT Radiation-induced TGF-β is one of main drivers leading to CAF-mediated fibrosis as well as the EMT program. Radiation-activating CAFs also promote TGF-β secretion and ECM remodeling. ECM stiffness causes tumor cell dissemination via EMT progression. TGF-β induces metastatic dormancy through EMP in distant organs. Targeting TGF-β is one of the best options to inhibit radiation-mediated metastasis.

Collectively, the current understanding of the relationship between radiation and metastatic dormancy is extremely limited. However, the strong fragmental lines of evidence below indicate that radiation can induce metastatic dormancy for long-term relapse: (1) radiation causes metastasis through EMT, (2) radiation induces TGF-β and CAFs, and (3) TGF-β activates EMP and metastatic dormancy. These possibilities indicate that a promising molecular target for RT-mediated metastasis is TGF-β. Theoretically, the blocking of TGF-β can inhibit both radiation-induced fibrosis via CAFs and radiation-induced EMT and metastatic dormancy (Figure 5).

The combination of RT and TGF-β inhibitors has been in clinical trials (Table 2). Fresolimumab, an anti-TGF-β antibody, combined with stereotactic ablative radiotherapy (SABR) reduces radiation-induce pulmonary fibrosis (NCT02581787). A higher dose of fresolimumab was found to be associated with longer median overall survival and increased immune cells such as central memory CD8^+^ T cells in metastatic breast cancer patients with RT (NCT01401062). Galunisertib, a TβRI kinase inhibitor, showed preliminary efficacy in patient with rectal cancer (NCT02688712). However, galunisertib failed to provide clinical benefit in patients with glioma (NCT01582269). The clinical trial combining vactosertib, a TGF-β receptor I inhibitor, and chemoradiation for esophageal adenocarcinoma is ongoing (NCT06044311).^117^^,^^118^^,^^119^^,^^120^

**Table 2:** Drugs to overcome radioresistance under ongoing clinical trials

Targets	Drug name	Disease	Clinical trials	Combination	Treatment	Reference
TGF-β	fresolimumab	non-small cell lung cancer	NCT02581787 (completed)	stereotactic ablative radiotherapy (SABR)	phase 1_fresolimumab 3 mg/kg I.V. on days 1, 15, and 36 and SABR in 4 fractions between days 8 and 12 phase 1_fresolimumab 1 mg/kg I.V. on days 1, 15, and 36 and SABR in 4 fractions between days 8 and 12phase 2_fresolimumab was administered I.V. at the dose selected in the preceding phase 1 on days 1, 15, and 36 and SABR was administered in 4 fractions between days 8 and 12	a
	fresolimumab	metastatic breast cancer	NCT01401062 (completed)	radiotherapy	phase 2_fresolimumab was administered intravenously (i.v.) at a dose of 1 mg/kg on day 1 of weeks 0, 3, 6, 9, and 12 and radiation administered at 7.5 Gy/fraction in 3 fractions during weeks 1 (to lesion 1) and 7 (to lesion 2); phase 2_fresolimumab was administered intravenously (i.v.) at a dose of 10 mg/kg on day 1 of weeks 0, 3, 6, 9, and 12, and radiation administered at 7.5 Gy/fraction in 3 fractions during weeks 1 (to lesion 1) and 7 (to lesion 2)	Deng et al.^117^
	galunisertib (LY2157299)	rectal cancer	NCT02688712 (active, not recruiting)	chemoradiation + surgery	phase 2_LY2157299 is administered for 14 days. On day 15, patients will begin chemoradiation treatment with capecitabine or fluorouracil. On day 29, patients will undergo another fourteen days course of LY2157299, concurrent with their ongoing chemoradiation treatment.	Formenti et al.^118^
	galunisertib (LY2157299)	malignant glioma	NCT01220271 (completed)	temozolomide-based radiochemotherapy	phase 1_ LY2157299 160 mg during radiation therapy:radiation: approximate 1.8–2.0 Gy × 30 fractions. approximate total dose = 60.0 Gy taken for 5 days per week for 6 weeks.LY2157299: 80 mg taken twice daily for 14 days followed by 14 days of pause; this on/off schedule constitutes a cycle of 28 days;temozolomide: 75 mg/m^2^ daily for 6 weeks after radiation therapy:LY2157299: 80 mg twice daily for 14 days followed by 14 days of pause; this on/off schedule constitutes a cycle of 28 days, taken for 6 cycles;temozolomide: 150 mg/m^2^ and then 200 mg/m^2^ daily during the off time of LY2157299, starting 28 days after the completion of radiation therapy; taken for 5 days followed by 23 days of rest for 6 cycles; phase 1_ LY2157299 300 mg; same as above treatment;phase 2_ LY2157299 selected in phase 1 and same as temozolomide and radiotherapy;	Yamazaki et al.^119^
	vactosertib	esophageal adenocarcinoma	NCT06044311 (recruiting)	chemoradiotherapy	phase 2_vactosertib orally, 200 mg twice daily for five days a week for 2 weeks, followed by standard-of-care chemoradiotherapy, followed by vactosertib for 4 weeks after standard-of-care chemoradiotherapy	a

a https://www.clinicaltrials.gov/

## Conclusions and future perspectives

The accumulating technology of RT and a better understanding of the effect of RT on TME are expanding the utilization of RT in clinically unmet needs. RT still exhibits limited efficacy for solid tumors due to its potential to cause radioresistance. In this review, we summarize the molecular mechanisms of radiation effects; radiation induces distant relapse via EMP, EMP-mediated dormancy, and metastasis. We address the possibility that TGF-β is one of the potential targets that prevent radiation-mediated metastatic cancer progression and suggest that targeting metastatic dormant cells will be a promising option to protect against metastasis after RT. However, the understanding of the triple relationships between RT, TGF-β-mediated EMP, and metastatic dormancy is almost completely lacking. To address this question, further detailed studies exploring the following are necessary: (1) whether RT controls TGF-β and induce metastatic dormancy via EMP, (2) whether RT causes cancer dormancy or awakens dormant cancer cells, and (3) whether metastatic dormancy is essential for RT-induced metastasis.

## Acknowledgments

This work was supported by grants from the 10.13039/501100008003Korea Institute of Radiological & Medical Sciences (KIRAMS, 50531-2026).

## Author contributions

Conceptualization, H.-Y.J.; data curation, Y.L. and H.-Y.J.; investigation, Y.L., B.J., and H.-Y.J.; visualization, H.-Y.J.; writing – original draft, H.-Y.J.; writing – review & editing, H.-Y.J.; funding acquisition, H.-Y.J.; project administration, H.-Y.J.; supervision, H.-Y.J.

## Declaration of interests

The authors declare no potential conflicts of interest.

## Declaration of generative AI and AI-assisted technologies in the writing process

Scientific content, data analysis, interpretation, and conclusions were developed by the authors. The authors used AI-assisted tools (EditGPT) only for grammar and language editing in the writing process.
